# Vascular endothelial effects of collaborative binding to platelet/endothelial cell adhesion molecule-1 (PECAM-1)

**DOI:** 10.1038/s41598-018-20027-7

**Published:** 2018-01-24

**Authors:** Raisa Yu. Kiseleva, C. F. Greineder, C. H. Villa, O. A. Marcos-Contreras, E. D. Hood, V. V. Shuvaev, H. M. DeLisser, V. R. Muzykantov

**Affiliations:** 1 Department of Pharmacology and Center for Translational Targeted Therapeutics and Nanomedicine of the Institute for Translational Medicine and Therapeutics, University of Pennsylvania, Philadelphia, PA United States of America; 2 Pulmonary, Allergy & Critical Care Division, The Perelman School of Medicine, University of Pennsylvania, Philadelphia, PA United States of America

## Abstract

Targeting drugs to endothelial cells has shown the ability to improve outcomes in animal models of inflammatory, ischemic and thrombotic diseases. Previous studies have revealed that certain pairs of ligands (antibodies and antibody fragments) specific for adjacent, but distinct, epitopes on PECAM-1 enhance each other’s binding, a phenomenon dubbed Collaborative Enhancement of Paired Affinity Ligands, or CEPAL. This discovery has been leveraged to enable simultaneous delivery of multiple therapeutics to the vascular endothelium. Given the known role of PECAM-1 in promoting endothelial quiescence and cell junction integrity, we sought here to determine if CEPAL might induce unintended vascular effects. Using a combination of *in vitro* and *in vivo* techniques and employing human and mouse endothelial cells under physiologic and pathologic conditions, we found only modest or non-significant effects in response to antibodies to PECAM-1, whether given solo or in pairs. In contrast, these methods detected significant elevation of endothelial permeability, pro-inflammatory vascular activation, and systemic cytokine release following antibody binding to the related endothelial junction protein, VE-Cadherin. These studies support the notion that PECAM-1-targeted CEPAL provides relatively well-tolerated endothelial drug delivery. Additionally, the analysis herein creates a template to evaluate potential toxicities of vascular-targeted nanoparticles and protein therapeutics.

## Introduction

Endothelial cells (ECs) play critical roles in the vasculature, sensing and responding to changes in blood flow, maintaining blood fluidity, and controlling the entry of leukocytes and plasma constituents into underlying tissues^1^. Cell adhesion molecules (CAMs), including Intercellular Adhesion Molecule-1 (ICAM-1)^2,3^, Platelet-Endothelial Cell Adhesion Molecule-1 (PECAM-1)^4–6^, Vascular Cell Adhesion Molecule 1 (VCAM-1)^3,7^, and both P- and E-selectins^3,7,8^, are involved in all of these functions. CAMs have been studied extensively as targets for affinity-mediated delivery of therapeutics to ECs, which are otherwise notoriously resistant to drug uptake^9^.

Collaborative Enhancement of Paired Affinity Ligands (CEPAL) is a recently described drug delivery paradigm, which allows cellular targeting of multiple drugs or nanoparticles through the use of antibodies (mAbs) or other protein affinity ligands directed to distinct, but adjacent, epitopes on a single surface molecule^10–13^. In CEPAL, the paired affinity ligands are not only non-competitive, but enhance each other’s binding. CEPAL has the potential to significantly expand the armamentarium of vascular drug targeting by enabling dual delivery of paired therapeutics within close enough proximity to allow their partnering at the cell surface or uptake into the same intracellular compartment. As an example, we showed that co-delivery of recombinant thrombomodulin (TM) and the endothelial protein C receptor (EPCR) by paired PECAM-1 specific fusion proteins markedly increased the activation of protein C *in vitro* and *in vivo* and decreased endothelial activation and vascular permeability in a murine model of acute lung injury^14^.

These results mandate careful investigation of potential unintended effects of CEPAL – particularly given the vital role of PECAM-1 in maintaining endothelial integrity and vascular barrier function^6^. While engagement of certain PECAM-1 epitopes has been shown to alter endothelial barrier function *in vitro*, little is known about the effects of binding of affinity ligands *in vivo*. Toxicity has been well-documented for antibodies to another junctional protein, vascular endothelial cadherin (VE-Cadherin), with a number of clones (BV9, BV6, BV13) shown to induce vascular permeability or even pulmonary hemorrhage in animals^15,16^.

The purpose of the current study was to define the effects of targeting PECAM-1 using paired epitopes identified in previous studies of CEPAL^12,14^. Emphasis was placed on measurement of effects *in vivo* and correlating them with *in vitro* assays using both mouse and human ECs, using VE-Cadherin antibody as positive control for validating the sensitivity of our methods. Moreover, key measurements were not only made in healthy animals and quiescent cells, but also in states of stress or disease, to better simulate the conditions in which PECAM-1-targeted anti-inflammatory, antioxidant, and antithrombotic therapeutics would presumably be applied. Taken together, these methods provide a preliminary blueprint for investigating potential toxicity arising from endothelial-targeted protein therapeutics and nanotechnology.

## Results

### Barrier function in mouse and human ECs

**Figure 1:**
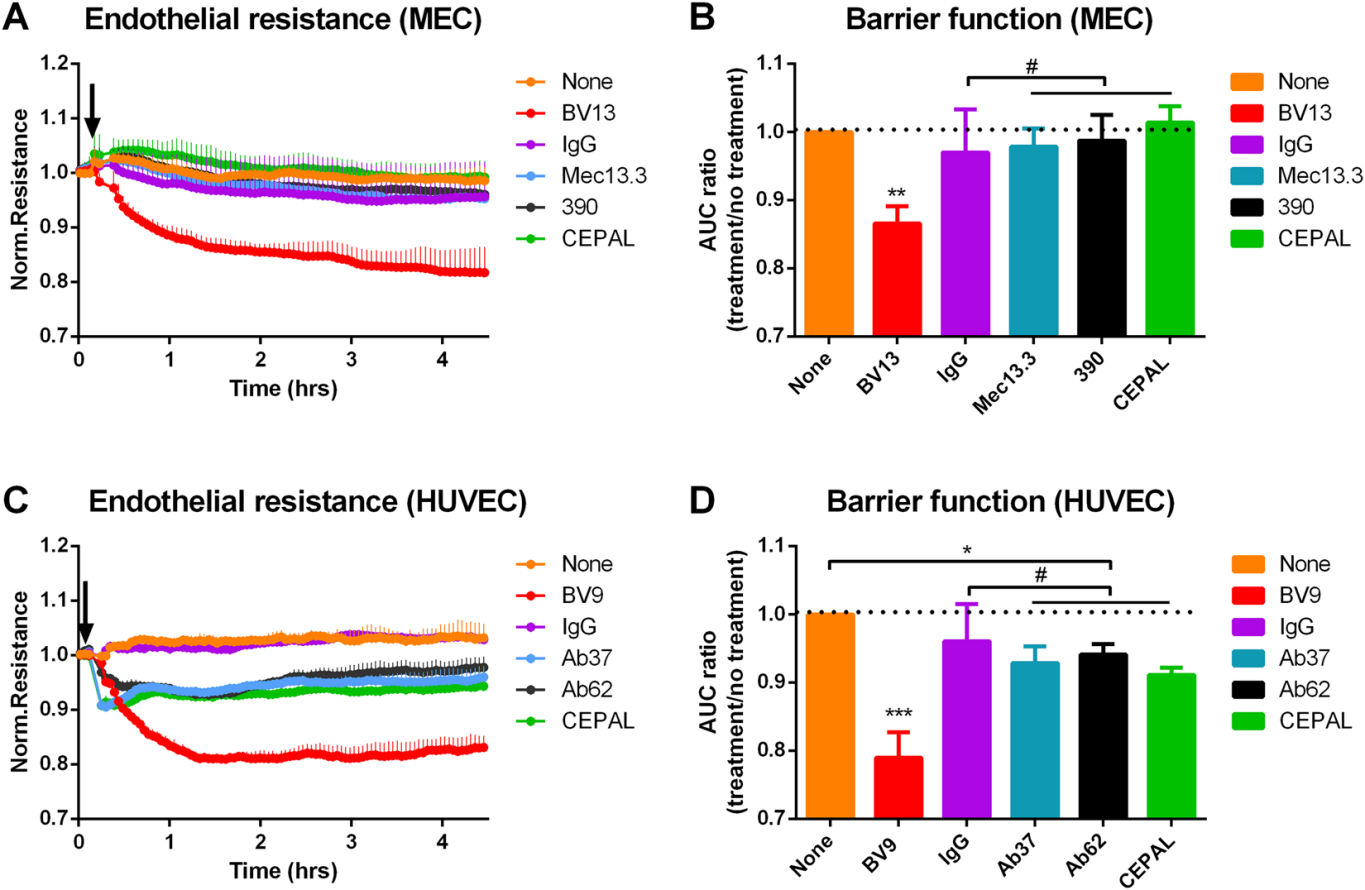
Barrier function assay in mouse and human endothelial cells. Electric cell-substrate impedance sensing (ECIS) measurement of endothelial resistance. (**A** and **C**) Real time tracings (representative of 4 independent monolayers) in MECs and HUVECs starting at the time of addition of specific antibodies or isotype controls. Presented resistance is normalized to a pre-treatment time-point for comparison of mAb effect on barrier function. (**B**) Quantification of AUC normalized to no treatment control. Anti-PECAM-1 antibodies had no effects on the electrical resistance of MEC monolayers in comparison to no treatment or isotype control (^#^p = 0.97, 0.99 for solo mAbs and 0.51 for a combination, respectively). Data shown as mean ± SD, dotted line illustrating a level of no treatment control. (**D**) In quiescent HUVEC cells, binding to PECAM-1, both solo and paired, results in significant drop in endothelial resistance (*p < 0.01, compared to no treatment). Effect mAbs to PECAM-1, when compared to IgG isotype control, was not significant (^#^p = 0.36 for Ab62 vs. mouse IgG2A and p = 0.81, for Ab37 vs. mouse IgG1). In both systems, significant barrier disruption was seen after binding of mAb to VE-cadherin (**p = 0.0005; ***p < 0.0001, compared to IgG isotype control). Data shown as mean ± SD, dotted line illustrating a level of no treatment control.

We first investigated the effects of collaborative binding to PECAM-1 on endothelial barrier function *in vitro*, using Electric Cell-substrate Impedance Sensing (ECIS) of cultured mouse EC (MEC) monolayers, which endogenously express both PECAM-1 and VE-cadherin. Antibodies were used at near-saturating concentrations (5–10 times the K_d_), matching our previous studies^12,13^. VE-cadherin specific antibody (BV13), our positive control for antibody-induced barrier disruption, induced sustained loss of transendothelial electrical resistance (TEER) (Fig. 1A). In contrast, neither anti-PECAM-1 clone (Mec13 or 390, 25 nM each) nor a mixture of the two antibodies compromised barrier function in these cells. Quantification (Fig. 1B**)** shows a significant drop in the ratio of the area under the curve (AUC) for antibody treated vs. non-treated control for BV13 (p = 0.0005), but not for any of the anti-PECAM-1 mAb groups (p = 0.97, 0.99, and 0.51 for Mec13.3, 390, CEPAL, respectively, compared to no treatment control).

We also measured the effects of anti-PECAM-1 mAbs in stably transfected REN cells (RmP), which express mouse PECAM-1 and form monolayers with electrical resistance near that of endothelial cells. In this case, both clones, 390 and Mec13.3, caused a drop in TEER (p < 0.005), with Mec13 inducing a significantly greater effect (p < 0.0001, Mec13.3 vs 390). CEPAL, induced by a mixture of the two antibodies, had no further effect, however, beyond that of the Mec13 mAb alone (p = 0.46) (Figure S2A**)**. Again, these results were quantified by calculating the ratio of AUC of the specific antibodies vs non-treated control (Figure S2B).

Finally, we investigated the effect of huPECAM-1 specific antibodies on transmembrane electrical resistance in human ECs (HUVEC). As with MECs, an anti-VE-cadherin antibody, clone BV9, was used as a positive control and induced a profound and sustained loss of TEER. In this case, each of the paired anti-PECAM-1 antibodies, clones Ab62 and Ab37, had a significant effect on barrier function (p = 0.001 and p = 0.008 vs. no treatment control), but this result was not different from isotype control (p = 0.36 for Ab62 vs. mouse IgG_2A_ and p = 0.81, for Ab37 vs. mouse IgG_1_). Once again, CEPAL – induced by exposure of cells to a mixture of the two antibodies (25 nM each) – had no effect on TEER beyond that of the individual antibodies (p = 0.87 and p = 0.43 in comparison to Ab62 and Ab37, respectively).

### Recovery of barrier function in thrombin challenged human ECs

**Figure 2:**
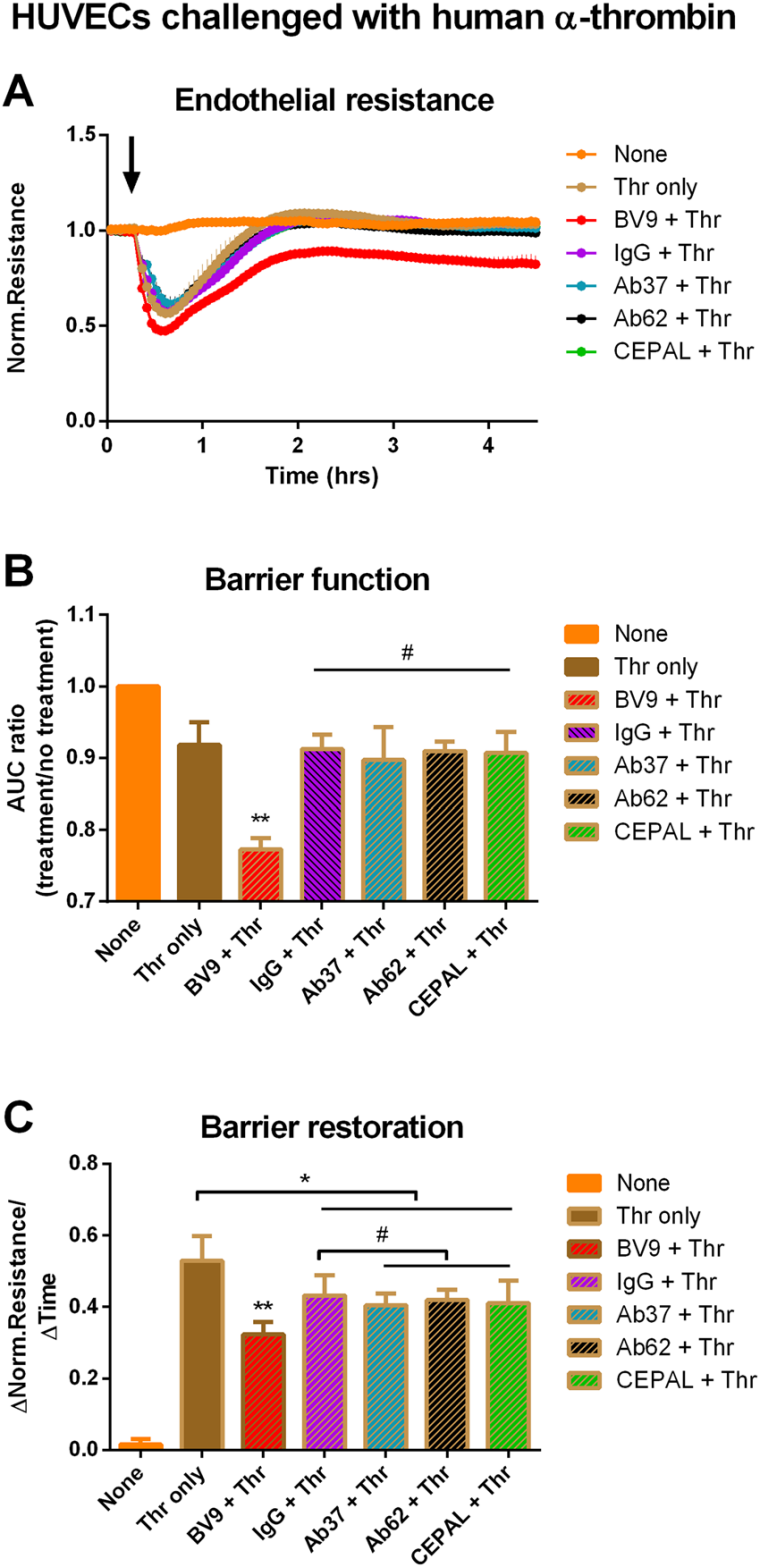
Barrier function restoration in human endothelial cells. (**A**) ECIS measurement of HUVECs pre-incubated with mAbs and challenged with human α-thrombin. Data shown are mean ± SD for 4 independent monolayers. (**B**) Ratio of area under the curve (AUC) of antibody-treated vs. no treatment control. Anti-PECAM-1 antibodies (Ab62 and Ab37 solo or paired) do not result in additional drop in endothelial resistance (^#^p = 0.99, 0.75, and 0.90, respectively), whereas anti-VE-cadherin antibody, clone BV9, exaggerates both the initial drop in permeability induced by thrombin (**p < 0.0001), and (**C**) barrier restoration, as reflected in the slope of recovery (**p < 0.0001). Anti-PECAM-1 antibodies slightly delayed barrier recovery but, unlike BV9, were not significantly different from isotype control (^#^p = 0.93, 0.99, and 0.90, respectively).

Having established a lack of effect on membrane permeability in resting endothelial monolayers, we next investigated if CEPAL would alter recovery of electrical resistance after thrombin-induced permeability. Similar results have been demonstrated for antibodies to other PECAM-1 epitopes, although this has not been studied for any of the clones known to demonstrate collaborative binding^17,18^. As shown in Fig. 2A and B, neither Ab62 nor Ab37 nor their combination had any effect on TEER following thrombin exposure (p = 0.99, 0.75, and 0.90, respectively), whereas BV9 both exacerbated initial thrombin-induced loss of permeability and prevented restoration of initial monolayer TEER (p < 0.0001 vs. thrombin treatment only). Focusing solely on the recovery phase (Fig. 2C), all antibodies resulted in slight slowing of the recovery of TEER after thrombin treatment, calculated as the rate of change of electrical resistance between the nadir and full recovery from initial thrombin insult (0.8 to 1.5 hours) (p < 0.0001 vs. thrombin treatment only). Again, neither Ab62 nor Ab37 nor their combination were significantly different from isotype controls (p = 0.93, 0.99, and 0.90, respectively)

### Pulmonary vascular barrier function *in vivo*

**Figure 3:**
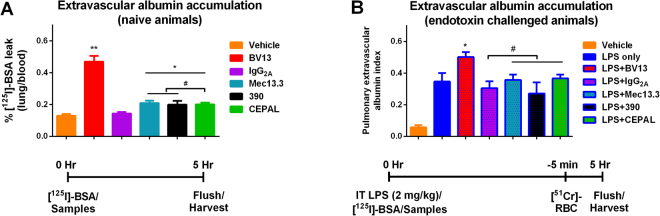
Extravascular albumin accumulation of radiolabeled albumin in lungs of naïve (**A**) and endotoxin challenged (**B**) mice. Samples of mAbs were injected intravenously just after injection of [^125^I]-BSA. (**A**) Anti-PECAM-1 mAbs 390 and Mec13.3 both cause significant elevation of BSA leakage in comparison to isotype control, IgG2A, as does the mixture of CEPAL antibodies (*p = 0.02, 0.04, and 0.04 for Mec13, 390, and CEPAL, respectively). Collaborative binding does not cause any additional effect, however, as compared to solo antibodies (^#^p = 0.76 and p = 0.95 vs Mec13 and 390, respectively). Anti-VE-Cadherin mAb, in contrast, caused significant vascular leak (**p < 0.0001, compared to IgG2A control). Data shown as mean ± SD. (**B**) Neither anti-PECAM-1 antibodies nor CEPAL mixture exacerbate vascular leakage in animals treated with intratracheal LPS (^#^p = 0.87, 0.23, and 0.74, respectively), whereas anti-VE-Cadherin results in further compromise of endothelial barrier function (*p = 0.02 compared to LPS + IgG2A control). Pulmonary extravascular albumin index (Equation 1) is calculated as described in Materials and Methods, with data shown as mean ± SD.

We next tested if collaborative binding of antibodies to PECAM-1 would disrupt endothelial barrier function *in viv*o. Radiolabeled albumin, used as a tracer of extravascular protein leakage, was injected just prior to antibodies and its lung accumulation was measured following flushing of the pulmonary vasculature 5 hours later. The dose of antibody, 100 µg/mouse, or 4.0 mg/kg, was chosen based on previous reports of the BV13 clone, which has maximal effect on pulmonary vascular permeability at this dose^19^. As shown in Fig. 3A, our data matched these previous reports, with marked pulmonary leakage of ^125^I-BSA following BV13 injection (p < 0.0001 vs IgG_2A_ control). The anti-PECAM-1 antibodies, Mec13 and 390, each produced modest, but statistically significant leakage of albumin, in comparison to IgG_2a_-injected animals (p = 0.02 and 0.04, respectively), but no additive effect was seen in the CEPAL group (p = 0.76 and p = 0.95 vs Mec13 and 390, respectively).

Similar experiments were conducted in mice in which inflammatory lung injury was induced via intratracheal injection of endotoxin (1 mg/kg). In these animals, flushing of the pulmonary circulation was found to be inconsistent, necessitating a dual isotope technique to determine pulmonary vascular leak^20^. The results (Fig. 3B) show a ~5-fold increase in the extravascular albumin index following LPS challenge. Neither Mec13.3 nor 390 nor their co-administration resulted in additional permeability beyond that induced by endotoxin exposure (p = 0.87, 0.23, and 0.74, respectively). In contrast, binding of VE-cadherin by BV13 had an additive effect, worsening the extravascular albumin index in LPS-challenged mice (p = 0.02, vs LPS + IgG_2A_ control).

### Pulmonary endothelial expression of inducible adhesion molecules

**Figure 4:**
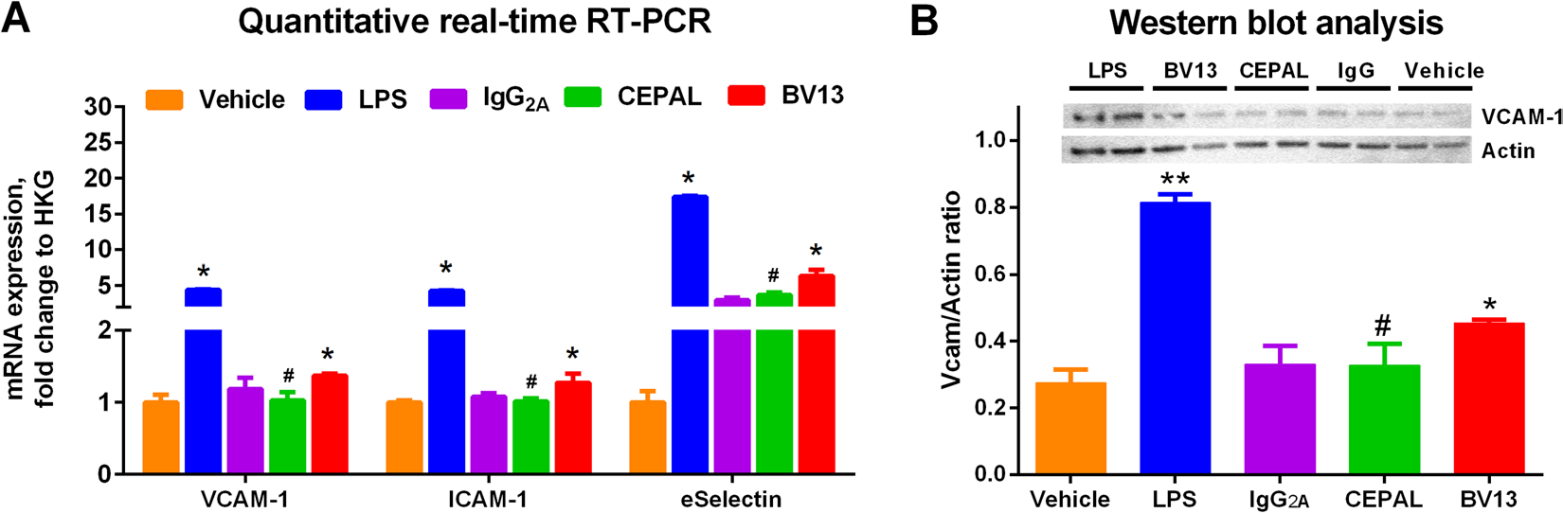
Expression of pulmonary activation markers in lung tissues. (**A**) Quantitative RT-qPCR revealed no significant increase in endothelial CAM expression in mice treated with paired anti-PECAM-1 antibodies, as compared to isotype control (^#^p = 0.99, 0.99, and 0.12 for VCAM-1, ICAM-1, and eSelectin, respectively). Anti-VE-Cadherin mAb, BV13, a positive control for antibody mediated barrier disruption, caused significant elevation of mRNA expression of all three CAMs (*p < 0.001 vs. isotype control). Graph shows mean ± SD for n = 3 animals per group. HKG – housekeeping gene. (**B**) Western blot showing lung homogenates (10 μg total protein/lane) stained for mouse VCAM-1 and α-actin. Quantification (VCAM/actin) using ImageJ software demonstrates an increase in VCAM-1 protein expression induced by LPS (**p < 0.001) and BV13 (*p < 0.01), but for CEPAL antibodies (^#^p = 0.28 vs. isotype control). Data presented as mean ± SD for n = 3 animals per group. Full-length western blot is presented in Supplemental Figure S3.

We next sought to determine if collaborative binding of paired antibodies to PECAM-1 might activate the pulmonary endothelium *in vivo*. Lung expression of a number of pro-inflammatory CAMs was tested five hours after injection of various antibodies or their isotype controls. As shown in Fig. 4A, anti-PECAM-1 mAbs (30 µg/mouse, or 2.4 mg/kg) did not significantly increase VCAM-1 or ICAM-1 mRNA over saline-injected controls (p = 0.99 for each). mRNA levels of a third adhesion molecule, E-selectin, were elevated in the CEPAL group in comparison to saline-injected controls (p < 0.0001), but not compared to mice treated with isotype control antibody (p = 0.12). BV13, in contrast, induced a significant increase in expression of all three adhesion molecules (p < 0.001 vs. isotype control).

We chose one adhesion molecule, VCAM-1, to confirm that changes in mRNA correlate with protein expression. Figure 4B shows western blotting of lung homogenates from animals treated with various antibodies. The combination of Mec13 and 390 had no significant effect, in comparison to IgG_2A_-injected animals (p = 0.28), whereas pulmonary VCAM-1 was elevated following BV13 (p < 0.01). As expected, endotoxin challenge (intravenous injection of LPS O111:B4, 2.5 mg/kg) caused a profound elevation of pro-inflammatory CAMs in the pulmonary vasculature, shown in both mRNA expression (p < 0.001 vs. IgG treatment control for all three CAMs) and VCAM-1 protein levels in lung homogenates (p < 0.001 vs. IgG treatment control).

### Systemic cytokine release

**Figure 5:**
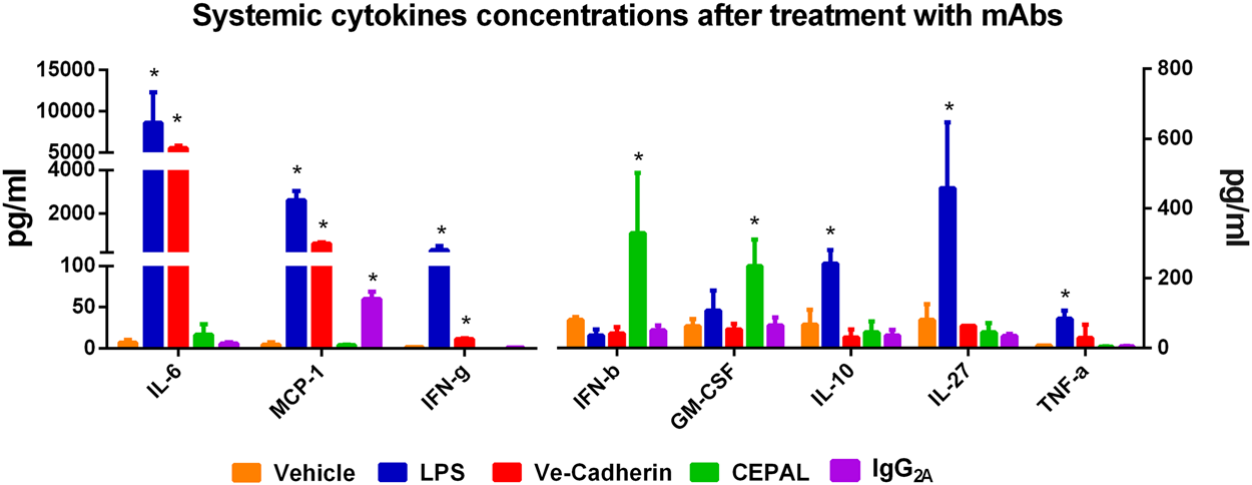
Systemic cytokine release *in vivo*. Cytokine concentrations from plasma samples of mice treated with mAbs were determined with the LEGENDplex Mouse Inflammation Panel (Biolegend). CEPAL mAbs cause significant increase in only two cytokines, GM-CSF and IFN-β (*p < 0.05, compared to isotype control). Results shown as mean ± SD. Complete dataset presented in Supplemental Table S1.

Studies conducted using PECAM-1 deficient mice have shown increased levels of inflammatory cytokines in a variety of animal models, indicating that PECAM-1 may have a role in regulating systemic inflammation^5,21^. To determine if CEPAL mAbs might affect cytokine release, we collected plasma from mice five hours after administration of various antibodies (~2.4 mg/kg total mAb) and tested for a panel of cytokines (IL-1α, IL-12p70, IL-1β, IL-17A, IFN-β, GM-CSF, IL-10, IL-27, TNF-α, IL-6, MCP-1, IFN-γ). Plasma from mice challenged with intravenous endotoxin (2.5 mg/kg) was used as a positive control. As shown in Fig. 5, collaborative binding to PECAM-1 had no effect on most cytokines, when compared to relevant controls, with only GM-CSF (p < 0.001) and IFN-β (p = 0.005) showing significant elevations. BV13, in contrast, induced release of multiple pro-inflammatory cytokines – IFN-γ, MCP-1 and IL-6 – with levels of the latter two comparable to those seen in LPS treated mice. Data for cytokine levels in endotoxin challenged mice obtained in this study were comparable with those described previously^22,23^.

## Discussion

PECAM-1 is a multifunctional cell adhesion molecule involved in numerous physiologic processes within the vasculature, including leukocyte adhesion and transmigration, angiogenesis, and maintenance of the transendothelial permeability barrier^6^. Many of its function are dependent upon formation of homodimers, which concentrate the protein at cell-cell junctions through a process called “diffusion trapping”^24^. Engagement of the extracellular domains of PECAM-1 by bivalent mAbs can perturb the dynamic equilibrium between PECAM-1 monomers, dimers, and oligomers, directly compromising barrier function or leading to bidirectional (outside-in and inside-out) signaling, with downstream effects on endothelial cell survival, junctional integrity, integrin expression, and leukocyte and platelet adhesion.

The numerous functions of PECAM-1 and their modulation by antibody binding mandate careful investigation of the possible adverse effects of employing this highly expressed, pan-endothelial molecule as a target for vascular drug delivery. Indeed, while nearly two decades of research have established the potential value of PECAM-1 targeted drugs and drug carriers^14,25–32^, toxicity and safety have yet to be investigated. Dual targeting applications using paired antibodies to PECAM-1 raise particular concerns about EC activation and vascular leak, given the conformational change and exposure of partially occult epitopes which underlie the collaborative binding phenomenon. With this in mind, probing the potential adverse effects of CEPAL to PECAM-1 became the focus of our first foray into this important subject.

The current study revealed a number of unexpected results. While each of our paired mouse-specific clones elicited a small, but statistically significant, increase in extravascular accumulation of albumin (1.42-fold, compared to isotype control), collaborative binding had no additive effect. Moreover, these increases in permeability were small compared to that seen with the VE-cadherin-specific BV13 antibody^19^. The relatively modest effects were somewhat surprising, in light of previous reports suggesting a critical role for PECAM-1 in junctional integrity and modulation of barrier function by binding of Fab fragments to PECAM-1 on ECs^18,33^. Our findings could be explained by the particular epitopes bound by paired CEPAL antibodies, which do not appear to disrupt homodimer formation^13^. Alternatively, it could be that *in vivo*, engagement of PECAM-1 – even by more than one antibody – is not sufficient to cause significant pulmonary vascular leak. While these data are encouraging for the prospects of CEPAL-based therapies, we felt it was necessary to confirm them in the setting of coincident lung pathology, given the putative clinical use of PECAM-1 targeted therapeutics. In these animals, the effects of anti-PECAM-1 mAbs could not be appreciated, even with the more sensitive dual isotope measurement, most likely due to being “drowned out” by the substantial effect of LPS alone. The fact that the assay was adequate to detect an additive effect of anti-VE-Cadherin mAb, however, suggests that lack of sensitivity of the measurement was not a major concern.

These *in vivo* results were matched overall by ultra-sensitive measurements of transendothelial electrical resistance (TEER) made with the ECIS device. The greater sensitivity of this technique provided more nuanced distinctions – e.g., showing that MECs did not respond to anti-PECAM mAbs, whereas HUVECs did. These observations may reflect important differences in the role of PECAM-1 in distinct organisms or vascular beds. Overall, however, the finding of most significance was that CEPAL did not impact endothelial barrier function beyond the effects of solo antibodies or affect the recovery of TEER following thrombin induced challenge.

We hypothesized that in addition to disturbance of vascular barrier function, the collaborative binding to PECAM-1 at high dose of paired ligands might, in theory, also lead to other unintended consequences, including endothelial expression of pro-inflammatory CAMs and systemic release of pro-inflammatory cytokines. These hypotheses were again based on indirect evidence, either from *in vitro* studies of antibody binding to PECAM-1 on cultured ECs or animal studies involving PECAM-1 deficient mice^34–38^. Collectively, our results indicate that these toxicities are not a major concern following collaborative antibody binding to PECAM-1 *in vivo*. The relative lack of adverse effects in our studies can be explained by a number of factors. Even at moderate doses, the endothelium is not exposed to high blood concentrations of anti-PECAM-1 antibodies for prolonged periods following intravenous injection due to rapid target-mediated disposition (only ~40% of the injected dose remains in the blood at 30 minutes^12^). Alternatively, the presence of physiologic blood flow may have significant crosstalk with antibody engagement of PECAM-1, which is itself a component of the endothelial mechanosensory complex^39^, and could negate some of the effects seen in other experimental settings. Of note, CEPAL antibodies did result in significant elevations of two pro-inflammatory cytokines, GM-CSF and IFN-β, both of which play significant roles in angiogenesis^40,41^. While of unclear significance, these cytokines and pro-angiogenic pathways will require close examination in future translational studies involving CEPAL-based therapies.

In conclusion, we report a detailed assessment of the vascular endothelial effects of collaborative antibody binding to PECAM-1. While this novel drug delivery paradigm holds great promise for the development of endothelial-targeted therapeutics, increased understanding of its potential toxicities will be key to successful translation. Moreover, the approaches presented may serve as a blueprint for future studies of the potential adverse effects of vascular-targeted protein therapeutics and nanoparticles currently under investigation.

## Materials and Methods

### Reagents and antibodies

Human alpha-thrombin (“thrombin”) was purchased from Haematologic Technologies (Essex Junction, VT). Bovine Serum Albumin and lipopolysaccharide (LPS) from Escherichia coli (serotype O111:B4; O55:B5) were purchased from Sigma-Aldrich (St. Louis, MO).

QuantiTect primers for qPCR quantification of gene expression of mouse VCAM-1 (cat.# QT00128793), ICAM-1 (cat.# QT00155074), and E-selectin (cat.# QT00114336) were purchased from Qiagen (Hilden, Germany), along with the housekeeping genes beta-actin (cat.# QT01136773), tubulin (cat.# QT00159299), and HPRT (cat.# QT00166768).

Antibodies (anti-VE-cadherin monoclonal antibodies clones BV13, BV9; anti-mouse PECAM-1 monoclonal antibody Mec13; rat IgG_2a_, mouse IgG_1_ and IgG_2a_ Isotype controls) were purchased from Biolegend (San Diego, CA). Anti-mouse PECAM-1 monoclonal antibody 390 (mAb390, rat, isotype IgG2a)^42^ and anti-human PECAM-1 monoclonal antibodies clones 37 and 62 (mAb 37 and 62, mouse, isotypes IgG_1_ and IgG_2a_, respectively)^43^ were produced from parental hybridoma cell lines and purified from cell supernatant using a protein G column (GE Healthcare). Antibody purity was confirmed by SDS-PAGE and/or size exclusion HPLC (Yarra SEC-3000, LC column 300 × 21.2 mm). Goat polyclonal anti-mouse VCAM-1 and anti-goat- HRP antibodies were purchased from Santa-Cruz Biotech (Dallas, TX). Anti-beta- actin antibody conjugated to HRP was purchased from Abcam (Cambridge, MA).

### Cell culture

Unless otherwise indicated, cell culture reagents were purchased from Invitrogen (Carlsbad, CA). Human umbilical vein endothelial cells (HUVECs) were purchased from Lonza (Walkersville, MD) and maintained in EGM complete growth medium (Lonza). C57BL/6 Mouse primary lung microvascular endothelial cells (MECs) were obtained from the Cell Biologics (Chicago, IL) and maintained in Culture Complete Growth Medium (Cell Biologics). The human mesothelioma cell line, REN, and transfected REN stably expressing mouse PECAM-1 (REN-mPECAM, or RmP)^13^, were maintained in RPMI-Glutamax supplemented with 10% (v/v) fetal bovine serum, 1% (v/v) penicillin/streptomycin solution, and in case of transfected RmP 250 μg/mL G418 (Mediatech, Manassas, VA).

### Animals and Ethics Statement

Animal studies were performed in accordance with the Guide for the Care and Use of Laboratory Animals, as adopted by the National Institutes of Health, under protocols 805696 and 805708, approved by the University of Pennsylvania Institutional Animal Care and Use Committee. Male C57BL/6 mice weighing 20–30 g (Jackson Laboratory, Bar Harbor, ME) were used for all experiments. All mice were housed in a temperature and humidity controlled environment (18–23 °C with 40–60% humidity under a 12-hour light-dark cycle) with ad libitum access to food (Labdiet 5010 autoclavable rodent diet, Brentwood, MO) and water.

### Radiolabeling of BSA with [^125^I] and RBCs with [^51^Cr]

Bovine serum albumin was directly radioiodinated using [^125^I]NaI (Perkin Elmer, Waltham, MA) and pre-coated Iodination tubes (Thermofisher, Waltham, MA). Reaction mixtures were purified over a 2-mL Zeba Spin Desalting Column (7k MWCO; Thermofisher). Radiolabeling efficiency and radiochemical purity were assessed by the trichloroacetic acid assay and deemed acceptable if >95%.

The blood pool was labeled as previously described^44^. Briefly, blood was collected from CJ7BL/6 J mice into buffered sodium citrate and spun at 1000 × g for 10 min at 4 °C to isolate red blood cells (RBCs). Isolated RBCs were then resuspended to a 10% hematocrit with endotoxin-free normal saline and 1.5 mL aliquots were then incubated with 15 µL of a 1 mCi/mL solution of [^51^Cr] (GE Healthcare, Piscataway, NJ) for 2 h at room temperature. Labeled RBCs were washed 7 times with DPBS and re-suspended to a 10% hematocrit.

### Endothelial barrier function *in vitro*

Endothelial cell permeability was measured in real time using an Electric Cell-substrate Impedance Sensing (ECIS) Zθ device (Applied Biophysics, Troy, NY), following methods previously described by Keese and Giaever^45^. Each experiment was conducted at least four times on different days, using distinct cell preparations. Cell monolayers (MECs, RmP, or HUVECs) were grown on standard 8-well array (8W10E + PET) as described elsewhere^46,47^. Briefly, arrays were cleaned with L-cysteine, coated with 200 µl/well of warm 1% gelatin (100 ug/mL) (Sigma) for 30 minutes at 37 °C, washed with sterile water, and incubated with complete cell culture medium prior to seeding of cells (1*10^5^ cells/well). Monolayer impedance was monitored until EC confluence was verified as resistance reached a steady state. To model transient endothelial barrier disruption under pro-inflammatory/pro-coagulant conditions, cells were treated with human alpha-thrombin (0.5 U/well) for 1-hour post mAb treatment. Each treatment group was observed in triplicate. Data are presented as changes in the resistance normalized to its initial value at time-point when confluence is reached.

### Assessment of pulmonary endothelial activation

To assess pulmonary expression of pro-inflammatory CAMs following injection of various mAbs, lung tissues were harvested and prepared for Western blot or RT-qPCR. For the former, lungs were added with 1 ml of PBS supplemented with protease inhibitor cocktail and homogenized with 5 mm stainless steel bead using TissueLyser II (both are from Qiagen, Valencia, CA) during 6 min at 30 Hz. Tissue homogenate was further lysed by addition of 0.5% Triton X-100, 0.5% SDS (final concentrations) followed by incubation on rotating platform for 1 h at + 4 °C. Homogenates were sonicated with six 3-s strokes at 30% power using a Sonic Processor FB120 (Fisher) and centrifuged 10 min at 16,000 g. Supernatant was collected and protein concentration in the samples was measured by the DC Protein Assay (Bio-Rad, Hercules, CA). Samples were subjected on Ready gel 4–15% Tris-HCl (Bio-Rad). VCAM and active expressions were analyzed by Western blot using appropriate antibodies and VCAM level was normalized by actin. ImageJ was used for the analysis of densitometry results.

For measurement of mRNA levels using RT-qPCR, lung tissue (≤30 mg, at least 2 samples per animal) was homogenized in 600 μl of buffer RLT by high-speed shaking in plastic tubes with stainless steel beads using TissueLyser II (both are from Qiagen, Valencia, CA) during 6 min at 30 Hz. Total RNA was extracted using RNeasy Mini kit (Qiagen) following the manufacturer’s instructions. RNA concentration and purity were determined by NanoDrop3000 spectrophotometer (Thermo Fisher). Samples having spectrophotometric OD 260:280 ratios of 1.9–2.1 were used. Single stranded cDNA was synthesized using the High Capacity cDNA Reverse Transcription Kit (Applied Biosystems, Foster City, CA) and incorporated into RT-qPCR reaction mixtures in quadruplicates in a ViiA 7 Real-Time PCR System (Applied Biosystems). Reactions consisted of 2 μl of cDNA, 2 μl 10× primers (final concentration 0.2 μM), 10 μl of Fast SYBR® Green Master Mix (2×), and nuclease free Milli-Q® purified water to a total volume of 20 ul. Each cycle consisted of denaturation at 95 °C for 15 s, annealing at 58.5 °C for 5 s and extension at 72 °C for 10 s. Validated primers for the endothelial activation markers VCAM, ICAM, and E-selectin were used (Qiagen). Fold changes in mRNA were calculated according to the ΔΔCt method normalized to three housekeeping genes - beta-actin, tubulin, and HPRT^48^.

### Cytokine protein concentrations

For measurement of plasma cytokines following injection of various mAbs, blood samples were collected in heparin and spun at 1000 × g for 10 min at 4 °C. Cytokine levels were assessed using the LEGENDplex Mouse Inflammation Panel (Biolegend) according to manufacturer’s protocol. An Accuri™ C6 flow cytometer (Becton Dickinson, Franklin Lakes, NJ) was used to measure and LEGENDplex v.7.0 software was used to analyze the results.

### Extravascular albumin extravasation with radiolabeled albumin

Changes in pulmonary vascular permeability following injection of various mAbs were evaluated in accordance with the American Thoracic Society guidelines^20^. Specifically, barrier function was assessed by measuring the extravascular accumulation of [^125^I]-labeled albumin, injected intravenously in a tracer dose at the start of the experiment. At the experiment’s conclusion, blood was withdrawn from the inferior vena cava and animals were euthanized. A catheter was placed in the right ventricle to perfuse the pulmonary circulation. Following organ harvesting and weighing, the radioactivity of the blood and organs was measured with a 2470 Wizard2 gamma counter (PerkinElmer). In healthy animals, the localization ratio of [^125^I]-albumin, calculated as the (%injected dose present in lung/gram of lung tissue)/blood level), was used as a measurement of vascular leak.

In a separate group of experiments, designed to assess the effects of various mAbs in animals with acute pulmonary inflammation, extravascular accumulation of albumin was assessed using a dual-isotope technique^49^. Animals first received an intratracheal dose of endotoxin (O55:B5; 2 mg/kg) similar to that previously described^50^. LPS was resuspended in 50 μl of PBS and injected via a tracheal catheter placed by direct laryngoscopy, then immediately followed by 100 μl of air to ensure even distribution throughout all distal airspaces. Immediately after, animals were injected intravenously with various mAbs and [^125^I] -BSA and allowed to recover for 5 hours. Five minutes before the end of the experiment, animals were injected intravenously with 50 μl of [^51^Cr]-RBCs at 10% hematocrit to mark the blood pool. A blood sample was obtained from the inferior vena cava before the mice were euthanized by exsanguination and cervical dislocation. Following organ harvest and weighing, radioactivity was measured with a gamma counter. Calculations for dimensionless index of extravascular radioactive content were adapted from^49^ and determined as

1 $$Lung\,extravascular\,albu{\min }\,index=\frac{{[{}^{{125}}{\rm{I}}]}_{lung}-\frac{({[{}^{{125}}{\rm{I}}]}_{blood}\ast {[{}^{{51}}Cr]}_{lung})}{{[{}^{51}Cr]}_{blood}}}{W{W}_{lung}\ast \frac{{[{}^{{125}}{\rm{I}}]}_{blood}}{{W}_{blood}}}$$

where *[*^*125*^*I]*_*lung*_*, [*^*125*^*I]*_*blood*_*, [*^*51*^*Cr]*_*lun*g_, and *[*^*51*^*Cr]*_*blood*_ are the lung and blood counts per minute of each designated isotope; WW_lung_ is the wet weight (WW) of the lung sample in grams; and WB is the blood sample weight in grams. The activity of [^125^I]-BSA in the blood was included in the denominator to adjust for differing amounts of injected [^125^I]-BSA between mice.

### Data analysis and statistics

Results are expressed as means ± SD, unless otherwise noted. Statistical significance (p < 0.05) was determined by 1-way ANOVA with Dunnett’s or Bonferroni’s multiple comparison test as appropriate (Prism 6.0; GraphPad Software, La Jolla, CA).

### Data availability statement

All data generated or analyzed during this study are included in this published article (and its Supplementary Information files).

## Electronic supplementary material

Supplementary material
