# Supplementary material for: Vascular endothelial effects of collaborative binding to platelet/endothelial cell adhesion molecule-1 (PECAM-1)
Source: Sci Rep. 2018 Jan 24;8:1510. doi: 10.1038/s41598-018-20027-7 (PMC5784113; doi:10.1038/s41598-018-20027-7)
Supplement: Supplementary file 1 — Supplementary material [file 41598_2018_20027_MOESM1_ESM.pdf]

Vascular endothelial effects of collaborative binding to platelet/endothelial cell adhesion molecule-1 (PECAM-1)

Kiseleva R.Yu.*[1], Greineder C.F.*&[1], Villa C.H. [1], Marcos-Contreras O.A. [1], Hood E.D. [1], Shuvaev V.V. [1], DeLisser H.M. [1,2], Muzykantov V.R.[& 1]

[1]Department of Pharmacology and Center for Translational Targeted Therapeutics and Nanomedicine of the Institute for Translational Medicine and Therapeutics, University of Pennsylvania, Philadelphia, PA, United States of America

[2]Pulmonary, Allergy & Critical Care Division, The Perelman School of Medicine, University of Pennsylvania, Philadelphia, PA, United States of America

*Equal contribution

[&]Address correspondence to cgreineder@gmail.com; muzykantov@pennmedicine.upenn.edu

Table S1 Systemic cytokines levels measured in plasma of mice treated with mAb (pg/ml), presented as mean and SD.

	LPS		Ve-Cadherin		CEPAL		IgG_{2a}		Vehicle	
	Mean	SD	Mean	SD	Mean	SD	Mean	SD	Mean	SD
**IL-1α**	9.80	6.00	2.60	1.45	1.43	0.15	2.59	0.87	5.61	7.38
**IL-12p70**	10.57	1.80	2.02	0.22	1.65	0.11	1.69	0.16	1.15	0.08
**IL-1β**	48.23	9.61	25.02	10.51	8.62	0.83	18.69	4.03	17.10	1.79
**IL-17A**	7.19	1.72	6.05	0.48	2.93	0.10	2.23	0.25	1.22	0.10
**IFN-β**	36.05	16.89	41.55	18.70	329.53	172.20	50.49	14.31	80.02	9.32
**GM-CSF**	107.19	57.77	53.39	15.80	234.97	76.04	64.55	22.68	62.78	20.20
**IL-10**	241.47	39.95	30.53	22.82	44.64	30.61	35.93	15.74	66.51	43.40
**IL-27**	458.62	187.54	63.33	0.17	44.50	27.25	34.69	6.05	80.59	45.21
**TNF-α**	83.81	23.47	29.26	37.67	2.83	1.52	4.36	1.91	7.01	0.63
**IL-6**	8612.39	3677.38	5558.84	283.39	16.67	12.56	6.00	1.79	7.00	3.58
**MCP-1**	2617.37	426.40	625.70	52.05	4.00	0.89	60.00	8.94	4.67	2.88
**IFN-γ**	316.52	180.81	11.51	1.19	0.06	0.08	1.20	0.39	1.73	0.36

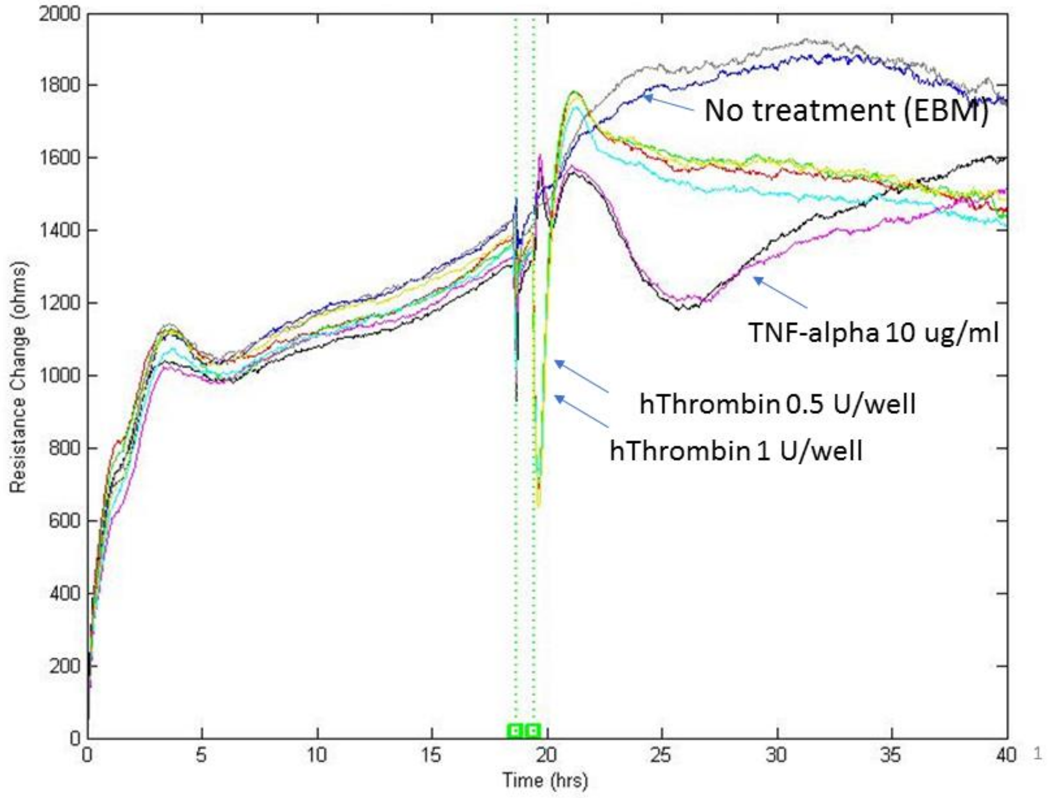

Figure S1. Representative real-time measurements of resistance in cell monolayers presented as a snapshot from ECIS software. Arrows indicate corresponding treatments. Detailed explanations of cell spreading and TEER read-outs can be found elsewhere (51).

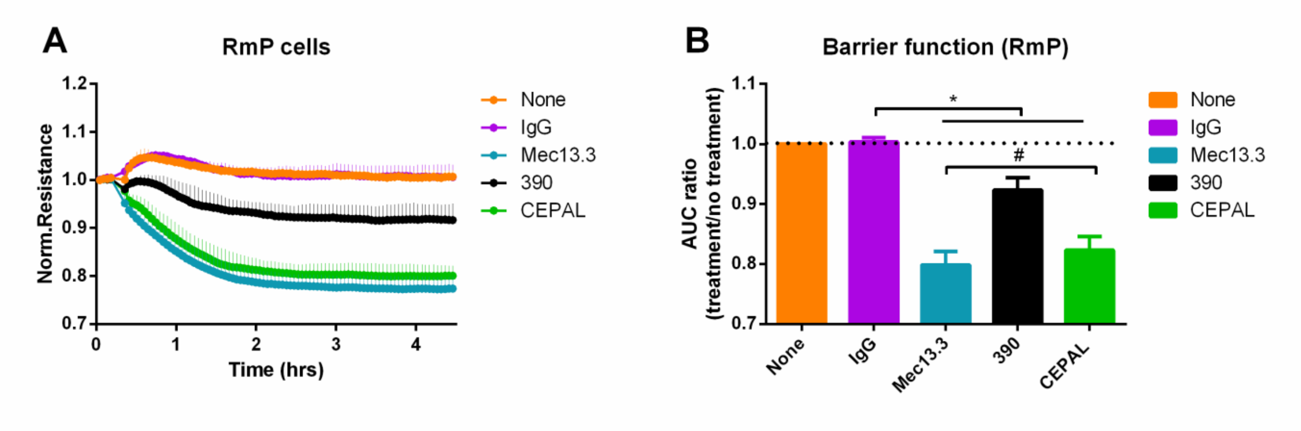

Figure S2. Electric cell-substrate impedance sensing (ECIS) measurement of RmP cells. A) Real time tracings (representative of 4 independent monolayers) starting at time of application of specific antibodies or isotype controls. Presented resistance is normalized to a pre-treatment time-point for comparison of mAb effect on barrier function. B) Quantification of areas under the curve (AUC) normalized to no treatment control. Both CEPAL (p<0.001) and solo anti-PECAM-1 mAbs, 390 (p=0.002) and Mec13.3 (p<0.001) induced significant barrier disruption in RmPs as compared to isotype control IgG$_{2a}$. The effect of Mec13.3 exceeded that of 390, but was not significantly enhanced by the combination of antibodies (#, p=0.46).

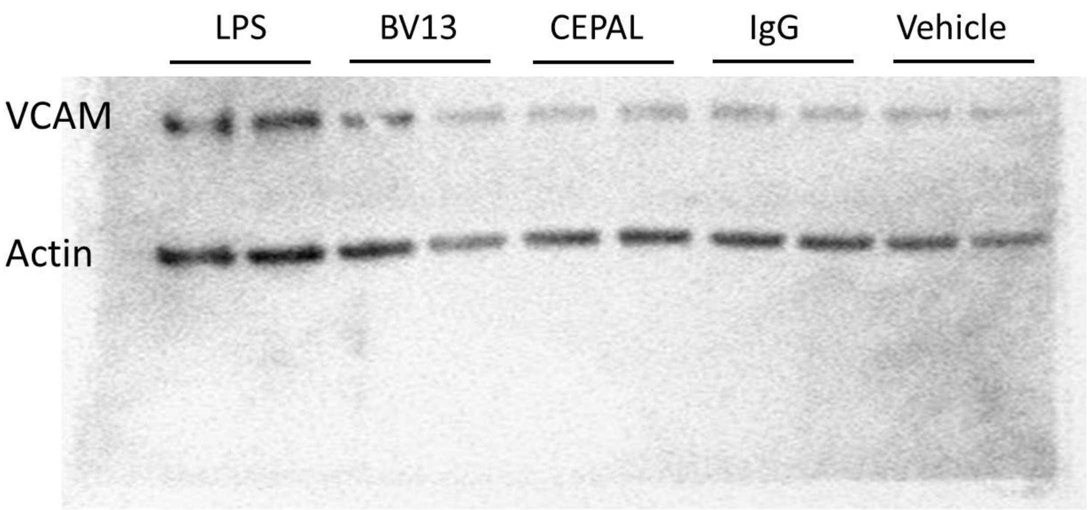

Figure S3. VCAM expression in lung homogenates. Full-length western blot showing samples (10 µg total protein/lane; representative image for n=2 in each group) stained for mouse VCAM-1. Actin was used as a loading control.
